# Posterior reversible encephalopathy syndrome secondary to acute post-streptococcal glomerulonephritis in a child: a case report from the Tibetan plateau

**DOI:** 10.1186/s12883-022-02750-x

**Published:** 2022-06-18

**Authors:** Bazhu, Yanhui Shi, Shuang Ren, Liang Shu, Qiang Li

**Affiliations:** 1 Department of Neurosurgery, Shigatse People’s Hospital, Tibet, China; 2 Department of Neurology, Xuhui District Central Hospital, Shanghai, China; 3 Department of Neurology, Shanghai Ninth People’s Hospital, Shanghai Jiao Tong University School of Medicine, 639 Zhizaoju Road, Shanghai, 200011 China

**Keywords:** Posterior reversible encephalopathy syndrome, Acute post-streptococcal glomerulonephritis, Children, Tibetan Plateau, Hyperbaric oxygen therapy

## Abstract

**Background:**

Posterior reversible encephalopathy syndrome (PRES) is a disorder of reversible vasogenic brain oedema with acute neurologic symptoms. It is a rare but serious disease that affects the central nervous system. PRES is a rare complication of acute post-streptococcal glomerulonephritis (APSGN). High altitude can accelerate vasogenic brain oedema by increasing cerebral blood flow (CBF), impairing cerebral autoregulation and promoting vascular inflammation. We report a case of PRES induced by acute post-streptococcal glomerulonephritis in a high-altitude environment.

**Case presentation:**

A fourteen-year-old Tibetan girl presented with progressive headache with haematuria, facial swelling, dizziness and vomiting for 2 weeks as well as multiple episodes of tonic–clonic seizures for 14 h. She was diagnosed with APSGN based on laboratory tests and clinical symptoms. Brain magnetic resonance imaging (MRI) and computed tomography (CT) revealed bilateral frontal, parietal and occipital lesions that were compatible with the radiological diagnosis of PRES. The treatments included an antibiotic (penicillin), an antiepileptic drug, and hyperbaric oxygen (HBO) therapy. Follow-up MRI obtained 1 week after admission and CT obtained 4 weeks and 6 weeks after admission demonstrated complete resolution of the brain lesions.

**Conclusions:**

The case illustrates a rare occurrence of PRES following APSGN in a 14-year-old child in the Tibetan Plateau. The hypoxic conditions of a high-altitude setting might lower the cerebral autoregulation threshold and amplify the endothelial inflammatory reaction, thus inducing PRES in patients with APSGN. It is important to recognize the clinical and radiologic features of PRES, and adjuvant HBO therapy can promote rapid recovery from this condition in high-altitude areas.

## Introduction

Posterior reversible encephalopathy syndrome (PRES) is a disorder of reversible vasogenic brain oedema with acute neurologic symptoms [1, 2]. It is a rare but serious disease that affects the central nervous system. Paediatric PRES cases seem to have a broader clinical and neuroradiological spectrum than adult cases. Childhood PRES is related to some underlying disorders, including renal diseases, systemic lupus erythematosus, sickle cell disease, and bone marrow or solid organ transplantation [2]. PRES is a rare complication of post-streptococcal glomerulonephritis in children. High altitude can accelerate vasogenic brain oedema by increasing cerebral blood flow (CBF), impairing cerebral autoregulation and promoting vascular inflammation [3, 4]. On the Tibetan Plateau, it is imperative to differentiate PRES from high-altitude cerebral oedema. Delayed diagnosis or misdiagnosis may lead to irreversible damage or even death. We promptly diagnosed a paediatric case of PRES induced by acute post-streptococcal glomerulonephritis in a high-altitude environment; the patient recovered rapidly after receiving antibiotic and antiepileptic treatment along with adjuvant hyperbaric oxygen (HBO) therapy.

## Case presentation

A 14-year-old Tibetan girl presented at the emergency department due to multiple episodes of tonic–clonic seizures for 14 h and progressive headache for 2 weeks with dizziness and vomiting. She reported a 1-month history of haematuria, fever and pharyngalgia. The patient had no history of hypertension, diabetes, head injury, or drug allergies. Her developmental and family history was normal. On admission, her blood pressure was 150/90 mmHg; physical examination showed somnolence, decreased muscle strength (grade 4/5) in all four limbs, and facial swelling.

**Fig. 1:**
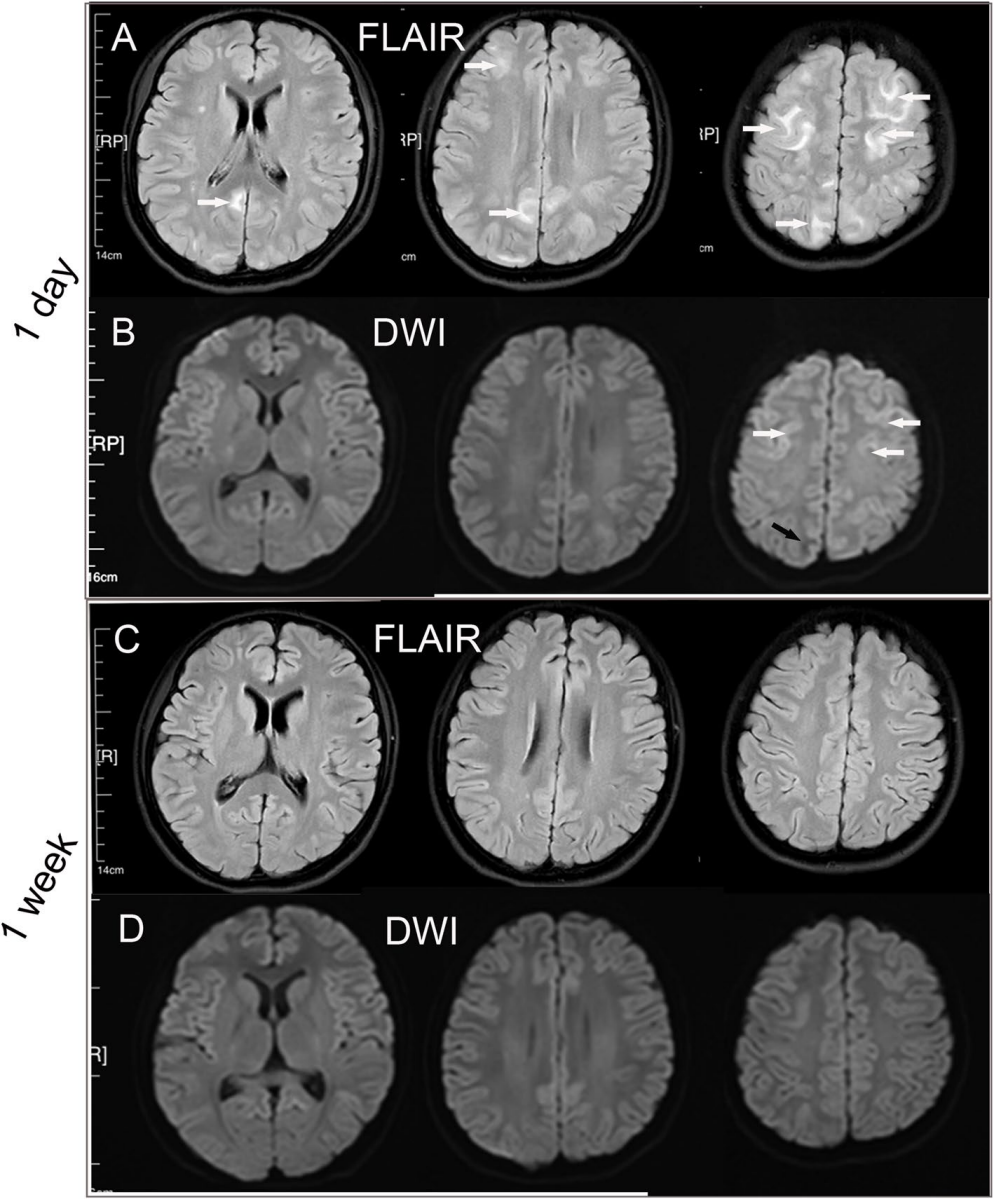
Initial and follow-up brain MRI of the patient. T2-FLAIR images obtained 1 day after the patient’s seizure show high-intensity signals in the bilateral frontal, parietal and occipital lobes (**A**, white arrow). DWI scans at 1 day show slightly hyperintense signals in the bilateral frontal cortex (**B**, white arrow) and hypointense signals in the right occipital lobe (**B**, black arrow). Follow-up T2-FLAIR (**C**) and DWI scans (**D**) at 1 week show resolution of the high-signal lesions

**Fig. 2:**
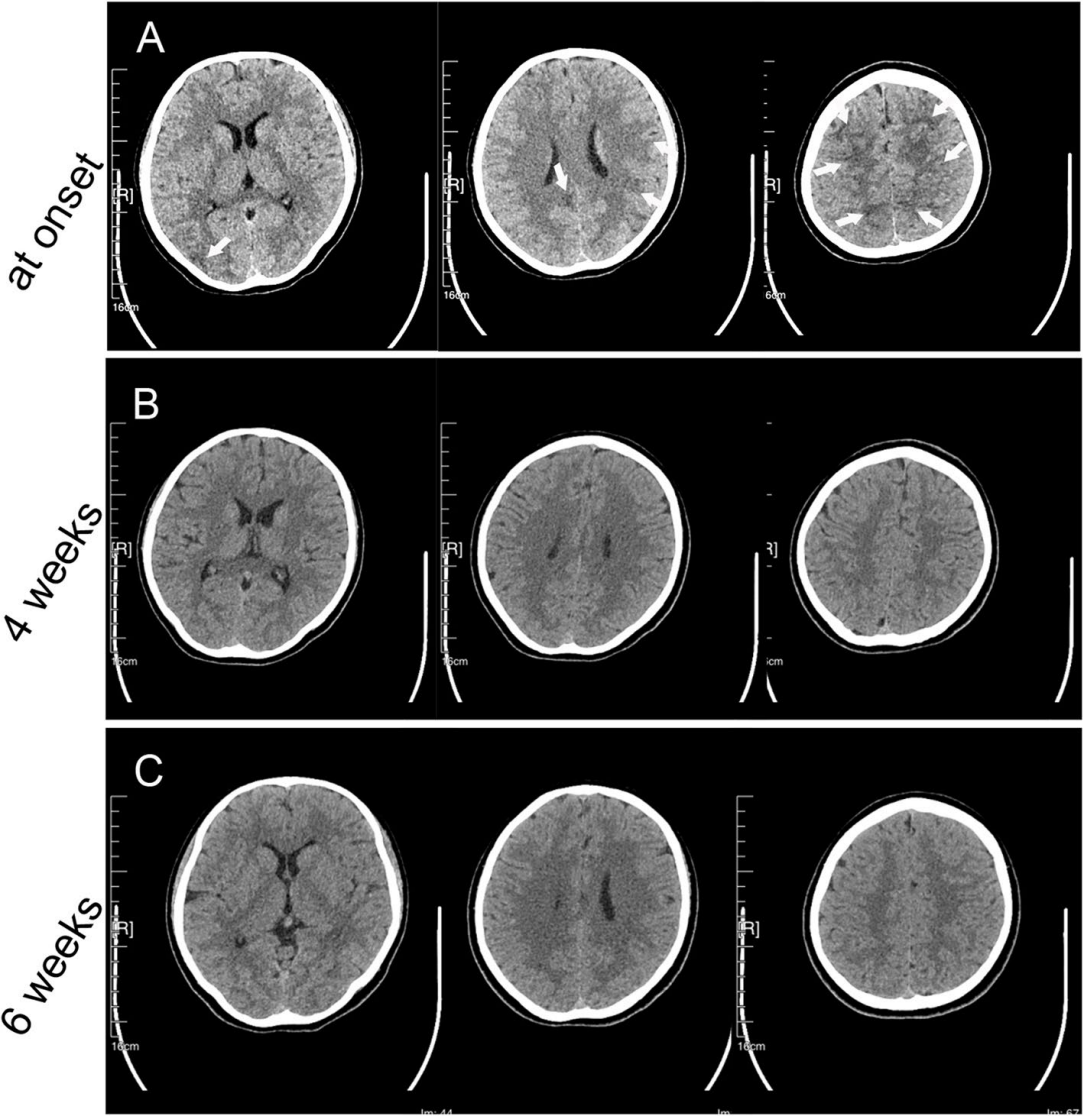
Initial and follow-up brain CT of the patient. Brain CT images at the onset of the patient’s seizure show hypointense signals in the bilateral frontal, parietal and occipital lobes (**A**, white arrow). Follow-up CT images at 4 weeks (**B**) and 6 weeks (**C**) after the patient’s seizure both show resolution of the hypointense lesions

**Fig. 3:**
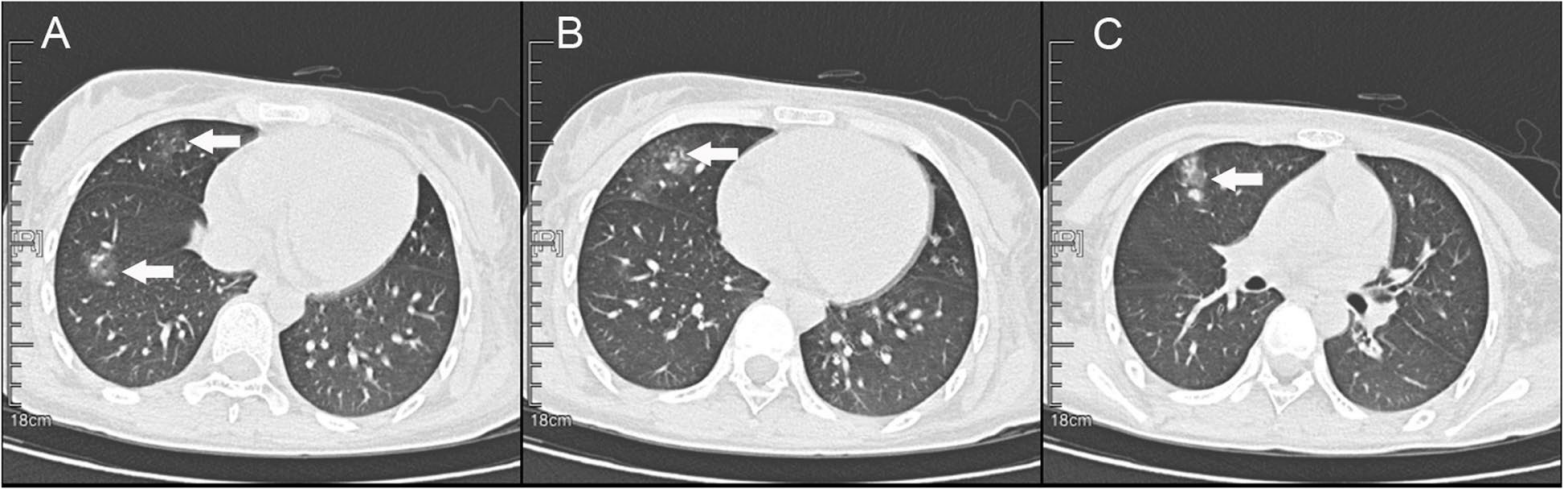
Initial chest CT of the patient. Chest CT images at the onset of the patient’s seizure show pneumonia in the right lung (**A**-**C**, white arrow)

At presentation, urinalysis identified significant haematuria (3 +), proteinuria (3 +), and mild leukocyturia (1 +). Blood laboratory tests (Table 1) showed the following remarkable results: azotaemia (urea 7.33 mmol/L, serum creatinine 168 μmol/L), infection (white blood cells 10 770 cells/mm^3^, C-reactive protein [CRP] 40.44 mg/L, hypersensitive CRP 10 mg/L), anaemia (haemoglobin 11.1 g/dL), increased anti-streptolysin O (ASO) antibody (890.4 IU/mL), and increased antinuclear antibody (ANA) (1:1280). Brain T2 fluid-attenuated inversion recovery (T2-FLAIR) images showed bilateral hyperintense signal changes in the frontal, parietal, and occipital lobes (Fig. 1-A). Diffusion-weighted imaging (DWI) showed slightly hyperintense signals in the bilateral frontal cortex and hypointense signals in the right occipital lobes (Fig. 1-B). Brain computed tomography (CT) revealed bilateral hypointense lesions in the frontal, parietal and occipital lobes (Fig. 2-A). A urinary ultrasound scan showed an enhanced echo in the tissue of both kidneys, and chest CT indicated pneumonia of the right lung (Fig. 3).

**Table 1:** Blood tests of the patient at onset and at 1 month

Variable	at onset	1 month
hCRP (N: 0–5 mg/L)	10.0	0.5
WBC (N: 3.5–9.5 × 10^9^/L)	10.9	5.54
Hemoglobin (N: 11.5–15.0 g/dL)	11.1	11.4
Urea nitrogen (N: 2.5–8.2 mmol/L)	7.33	3.73
Serum creatine (N: 22.0–132.0 μmol/L)	168.0	72.0
ASO (N:0–200.0 IU/L)	890.4	876.3
ANA (N: < 1:80)	1:1280	< 1:80
ds-DNA	(-)	(-)
p-ANCA	(-)	(-)
ACA	(-)	(-)
c-ANCA	(-)	(-)
β2-GP1-Ab	(-)	(-)
C3 (N: 0.8–1.6 g/L)	0.89	1.02
C4 (N: 0.2–0.4 g/L)	0.35	0.26

*Abbreviations*: *ACA* anti-cardiolipin antibody, *ANA* antinuclear antibody, *ANCA* antineutrophil cytoplasmic antibody, *ASO* anti-streptolysin O*, β2-GP1-Ab* β2-Glycoprotein 1 antibody, *C3* complement 3, *C4* complement 4, *hCRP* hypersensitive C reactive protein, *WBC* white blood cell count

A multidisciplinary team (MDT) composed of a neurologist, a nephrologist, a rheumatologist and a radiologist was involved in the patient’s care. Based on clinical symptoms and examination results, especially the acute onset of haematuria, acute kidney damage and proteinuria associated with increasing ASO, the patient was diagnosed with APSGN [5]. The typical cerebral MRI features of PRES include a bilaterally symmetrical parieto-occipital pattern, holohemispheric watershed pattern, and superior frontal sulcus pattern reflecting vasogenic oedema [6]. PRES was diagnosed on the basis of both an acute onset of multiple neurologic features and distinctive neuroimaging findings [1]. Differential diagnoses of ischaemic stroke, cerebral venous thrombosis, infectious encephalitis, autoimmune encephalitis, high-altitude cerebral oedema and central nervous system vasculitis were ruled out.

According to the opinions of the MDT, an antibiotic regimen (penicillin 6,400,000 U intravenously, twice a day for 2 weeks) was administered to treat pneumonia and acute post-streptococcal glomerulonephritis. An antiepileptic drug (sodium valproate, infused by a syringe driver for 3 days and administered orally for 2 weeks) was administered to control the seizures. HBO therapy (once per day, 1 ATA for 90 min) was given to prevent cerebral vasospasm, decrease cerebral oedema and protect the neurovascular unit. After 48 h of treatment, the patient had made favourable clinical progress; her seizures were controlled, and she regained full consciousness. Brain magnetic resonance imaging (MRI) after 7 days showed that the abnormal lesions had completely disappeared (Fig. 1-C, D). The patient was discharged on Day 14. At the 1-month follow-up, the patient’s facial swelling and haematuria had disappeared, her fatigue had improved, and her epilepsy had not relapsed. Repeated blood tests (Table 1) were normal except for mild anaemia (haemoglobin 11.5 g/dL) and increased ASO antibody (876.3 IU/mL). Brain CT after 4 weeks and 6 weeks showed normal results (Fig. 2-B, C). The patient and parents were highly satisfied with the care she had received in the hospital, and they expressed their appreciation to the department.

## Discussion and conclusions

PRES has been described previously as a very rare comorbidity of APSGN in children. This case is the first report of childhood PRES secondary to APSGN in a high-altitude area (over 4,000 m). In the present case, APSGN was diagnosed, with a previous upper respiratory tract infection, an elevated level of ASO antibody and acute kidney injury. In the context of APSGN, the patient developed headache and seizure associated with typical brain MRI findings of PRES. Based on the impact of high altitude, prompt adjuvant HBO therapy was administered, promoting rapid recovery; the clinical symptoms and neuroimaging abnormalities were resolved within a week.

The pathophysiology of PRES remains controversial, but most proposed explanations focus on the central role of hypertension. Hypertensive encephalopathy has traditionally been cited to explain this mechanism of PRES. Severe hypertension exceeds the limit of cerebrovascular autoregulation, leading to cerebral hyperperfusion and consequent extravasation of fluid and blood products into the brain parenchyma (vasogenic oedema) [6, 7]. Children seem to be more likely than adults to suffer from PRES under hypertensive conditions because the cerebral blood flow (CBF) autoregulation threshold is lower in children than in adults [2]. It has been suggested that aggressively lowering blood pressure can prevent pathological changes from benign vasogenic oedema to complicated cytotoxic oedema. In most reported cases of PRES with post-streptococcal glomerulonephritis [8, 9], blood pressure was severely elevated, thereby supporting the abovementioned theory. However, this patient’s blood pressure was 150/90 mmHg in the emergency room and consistently normal during hospitalization. Her moderate hypertension may be one of multiple concurrent causes, or it may be an epiphenomenon of PRES.

The pathophysiology of PRES is complex and diverse in this case. The immune response has played a significant role in the pathophysiology of this case. A wide variety of case reports have shown that PRES can occur in non-hypertensive patients due to eclampsia, severe infection, immunosuppressive medication, autoimmune diseases, or cancer chemotherapy [10, 11]. These PRES-related clinical conditions trigger the activation of the immune system, thus inducing endothelial dysfunction. A brain biopsy of a PRES patient provided evidence of endothelial activation, T-cell trafficking, and VEGF expression [12]. Another study provided evidence for the activation of innate immunity rather than adaptive immunity in the pathophysiology of PRES[13]. These reports all suggest that systemic immune system activation is involved in triggering PRES. Generally, in APSGN, the interactions of streptococcal antigens and antibodies induce immune system activation, including local complement activation, leukocyte infiltration and cytokine/chemokine production [14]. The immune system, when activated in APSGN, releases tumour necrosis factor (TNF)-α and interleukin (IL)-1β, which can upregulate the expression of vascular endothelial growth factor (VEGF), an important regulator of vascular permeability [14]. Therefore, immune system activation represents the key initial step in the pathogenesis of this case.

Notably, this case occurred on the Tibetan Plateau, at an altitude of more than 4,000 m. In this highland area, people are exposed to hypoxia. High altitude causes a reduction in barometric pressure and a consequent decrease in the partial pressure of oxygen (PO_2_), leading to deleterious effects. Some studies have demonstrated that natives of high-altitude locations develop increased blood viscosity, CBF and cerebrovascular responsiveness to adapt to the hypoxic environment [3]. Static CA is impaired in permanent high-altitude residents who live above 4,000 m [15, 16]. A previous study indicated that hypobaric hypoxia increased the levels of inflammatory cytokines, including TNF-α, IL-1β, and IL-6, in the plasma of humans and animals and induced cerebral oedema by upregulating AQP4 and TLR4 activation [17]. Moreover, high-altitude natives tend to show immune maladaptation when systemic immune responses are activated [18]. It is suggested that a high-altitude hypoxic environment lowers the cerebral autoregulation threshold and amplifies the endothelial inflammatory reaction, which plays a crucial role in the development of the disease.

The patient described in this report is a long-term Tibetan resident living at an altitude of over 4,000 m, where the oxygen content in the air is approximately 60% of that at sea level. Hypobaric hypoxia is a crucial characteristic of high-altitude areas. HBO therapy can improve the aerobic and neurochemical milieu in injured brain regions. HBO therapy has been shown to decrease cerebral oedema, attenuate inflammation, decrease apoptotic cell death, and promote neural regeneration in a variety of animal models [19]. In rats exposed to a high altitude, HBO therapy significantly inhibited the upregulation of IL-1β, IL-6, TNF-α, and IF-γ in brain tissue, and HBO therapy attenuated brain and pulmonary oedema by inducing HSP-70 expression [20]. Another study in minipigs with intracerebral haemorrhage (ICH) at high altitude demonstrated that HBO therapy mitigated blood–brain barrier disruption and suppressed the progression of brain oedema [21]. A clinical study on high-altitude ICH brain injury also reported that HBO treatment reduced brain oedema and tissue damage by improving cerebral oxygenation and metabolism [22]. During the management of our patient, in addition to baseline treatments such as antibiotic and antiepileptic therapy, HBO was given to improve focal cerebral metabolism, protect the neurovascular unit and attenuate inflammation. This strategy worked well for this case.

Seizures are reported to be the most common presenting symptom in the paediatric population, occurring in more than 90% of children [1, 2]. Generalized tonic–clonic seizures occur in approximately 60–75% of patients [6]. Although there are no studies available to guide the prescription of specific antiepileptic drugs, we recommend that antiepileptic drugs should be prescribed once the diagnosis of PRES is made. About the recurrent risk of seizures in PRES patients, retrospective studies suggest that few patients (10–15%) develop recurrent seizures in the first few years after PRES, most of which are attributed to provoking factors, including recurrence of PRES [23]. There are no prospective randomized studies to guide the optimum duration of treatment with antiseizure drugs. It might be appropriate to continue these drugs for several weeks after the initial presentation in patients without residual brain lesions [24].

In general, PRES with APSGN usually has a favourable prognosis if it is promptly diagnosed and treated. Past studies showed a full resolution of clinical findings, and neuroimaging lesions were documented from 3 to 16 weeks [25]. DWI can predict the prognosis of patients with PRES. Increased DWI signals in widespread areas are associated with neurological sequelae in PRES patients. In this case, DWI images at 1 day mainly showed hypo- to isointensity in lesion areas and slight hyperintensity in bilaterally frontal regions, which indicates major vasogenic brain oedema and minor cytotoxic brain oedema in the patient. Follow-up results also confirmed that the patient rapidly recovered, and all lesions disappeared within 1 week in this case.

There are several limitations to this case study. The active combination treatment promoted rapid improvements in this patient, but a study with a larger sample size should be launched to clarify the relationship of HBO intervention in particular with patients’ outcomes. Another potential limitation of the approach used in this study is the absence of magnetic resonance angiography (MRA), magnetic resonance venography (MRV) and MRI at the 1-month follow-up.

## Conclusions

The above case report illustrates a rare occurrence of PRES secondary to APSGN in a 14-year-old child in the Tibetan Plateau. There are few reported cases of PRES in high-altitude areas, perhaps owing to a lack of understanding of the disease. High-altitude hypoxic conditions might lower the cerebral autoregulation threshold and amplify endothelial inflammatory reactions, thus inducing PRES. Adjuvant HBO therapy can promote rapid recovery from PRES in high-altitude areas.

## Data Availability

All the data generated or analyzed during this study are included in this article, which are available from the corresponding author upon reasonable request.

## Abbreviations

ANA: Antinuclear Antibody
APSGN: Acute Post-streptococcal Glomerulonephritis
ASO: Anti-Streptolysin O
CA: Cerebral Autoregulation
CBF: Cerebral Blood Flow
CRP: C-Reactive Protein
CT: Computed Tomography
DWI: Diffusion-Weighted Imaging
HBO: Hyperbaric Oxygen
ICH: Intracerebral Haemorrhage
IL-1β: Interleukin-1β
MDT: Multidisciplinary Team
MRA: Magnetic Resonance Angiography
MRI: Magnetic Resonance Imaging
MRV: Magnetic Resonance Venography
PRES: Posterior Reversible Encephalopathy Syndrome
PO_2_: Pressure of Oxygen
T2-FLAIR: T2 Fluid-attenuated Inversion Recovery
TNF-α: Tumour Necrosis Factor-α
VEGF: Vascular Endothelial Growth Factor
